# Clinical Effect of Preservation or Nonpreservation of Left Colic Artery in Total Mesorectal Excision under Laparoscopy: A Meta-analysis

**DOI:** 10.1155/2020/1958573

**Published:** 2020-05-21

**Authors:** Jiefeng Liu, Yujing Gong, Miao He, Xinyu Zeng, Yiping Liu

**Affiliations:** ^1^Department of General Surgery, The Fourth Hospital of Changsha, Hunan Normal University, Changsha, 410006 Hunan Province, China; ^2^Department of Oncology, Xiangya Hospital, Central South University, Changsha, 410078 Hunan Province, China

## Abstract

**Background and Aims:**

To investigate the clinical effect of preservation or nonpreservation of the left colic artery (LCA) in total mesorectal excision (TME) under laparoscopy.

**Methods:**

The words, like “rectal cancer,” “left colonic artery,” and “laparoscopy,” were used as the retrieval terms, and the keyword retrieval method was adopted. The retrieval period was set as from January 1, 2013, to June 1, 2018. We searched databases including PubMed, Web of Science, and China National Knowledge Infrastructure (CNKI) to collect randomized and controlled trials which compared the effect of preservation or nonpreservation of the LCA in TME under laparoscopy. Two researchers independently carried out literature screening, data extraction, and literature quality evaluation; Review Manager 5.3 was used for the meta-analysis.

**Results:**

Seven studies including 1467 cases were identified for the meta-analysis. As showed by the meta-analysis, compared with the LCA nonpreservation group, the LCA preservation group had significantly reduced incidence of anastomotic leakage (OR = 0.44, CI = [0.30, 0.65], *P* < 0.0001) and postoperative urinary and sexual dysfunction (OR = 0.26, CI = [0.09, 0.78], *P* = 0.02) and significantly shorter time for intestinal function recovery (WMD = −0.26, CI = [−0.41, −0.11], *P* = 0.0008). There were no significant differences between the two groups in the duration of surgery, blood loss, number of dissected lymph nodes, or postoperative hospital stay.

**Conclusions:**

From the results, the LCA preservation group seems to achieve comparable success with acceptable safety outcomes. Therefore, this surgical method can be recommended in the clinical practice.

## 1. Introduction

Colorectal cancer is the third most common malignancy that causes significant morbidity and mortality in the world. More than 1.3 million people are diagnosed with colorectal cancer each year, and more than 600,000 patients die from colorectal cancer or related complications [1, 2]. At present, surgical treatment is still the main method for the treatment of rectal cancer. In 1908, Professor Miles first proposed low ligation, that is, resection of rectal cancer with the left colonic artery (LCA). In the same year, Professor Moynihan proposed the concept of high ligation, which was to ligature the inferior mesenteric artery by ligation at the distal end of the inferior mesenteric artery [3]. In recent years, with the development of the TME concept and the development of laparoscopic surgery, the surgical treatment of rectal cancer has undergone tremendous changes. In laparoscopic rectal cancer TME, the treatment of IMA and its branches mainly includes “high ligation and low ligation.” According to the American Association of Colorectal Surgeons guidelines, high ligation is ligation at the root of the IMA and does not retain LCA. Ligation at the lower site is ligation over the LCA branch of the IMA and retains the LCA [4, 5]. The branch of the inferior mesenteric artery (IMA) should be clearly dissected during the surgery, but there is a controversy over whether the LCA should be preserved [6]. Foreign researches in this aspect basically focused on in-hospital cases. The cases were compared for the duration of surgery, blood loss, number of dissected lymph nodes, incidence of postoperative anastomotic leakage, time for intestinal function recovery, postoperative urinary and sexual dysfunction, and recurrence after two years. Statistics on the incidence rate and overall survival were collected. In these independent studies, the number of cases collected was relatively small, which led to reduced reliability in the conclusion. Therefore, based on the existing researches, we conducted a meta-analysis on the published literature from January 01, 2013, to June 1, 2018, to investigate the effect of preservation of the LCA during laparoscopic TME for the treatment of rectal cancer. The clinical impact of arteries provides a reliable scientific basis for postoperative recovery of patients undergoing laparoscopic TME for the treatment of rectal cancer.

## 2. Data and Methods

### 2.1. Literature Retrieval Strategy

We search for laparoscopic rectal cancer surgery (using TME with or without the preservation of the LCA)-related articles published from January 01, 2013, to June 1, 2018, from databases including CNKI, PubMed, and Web of Science. Keywords retrieved were laparoscopic, rectal cancer, left colonic artery, rectal, laparoscopy, and left colic artery.

### 2.2. Inclusion Criteria

Inclusion criteria are the following: (1) literature study of the diagnosis of rectal cancer and laparoscopic TME for the treatment of rectal cancer; (2) literature with integral analytical data and independent studies including at least one control group with consistent purposes; (3) preservation or nonpreservation of the LCA as the only difference between the experimental group and the control group; (4) similar literature research methods; and (5) the combined results expressed by corresponding statistical indicators.

### 2.3. Exclusion Criteria

Exclusion criteria are the following: (1) duplicate or multiple articles about the same study, which may lead to content bias; (2) abstracts, research protocols, letters, editorials, comments, guidelines, and case reports; (3) and noncomparative studies.

### 2.4. Research Screening

Two researchers used a unified retrieval strategy to independently screen and extract data based on the inclusion and exclusion criteria. If there was a discrepancy, the decision shall be made through discussion or consultation with a third researcher. In this study, 37 related articles were obtained, among which 7 articles that met the inclusion criteria were finally included.

### 2.5. Statistical Analysis

A statistical analysis was performed on the retrieved domestic and foreign literature for the following: duration of surgery, blood loss, number of dissected lymph nodes, postoperative anastomotic leakage, time for intestinal function recovery, postoperative urinary and sexual dysfunction, two-year recurrence rate, and overall survival. The data from each article was compiled in a table and entered into a computer. The meta-analysis software Review Manager 5.3 was used to calculate and analyze the data. Weighted mean difference (WMD) and binary data were calculated for continuous variables. Odds ratio (OR) and combined values were expressed in 95% confidence interval (CI); the heterogeneity test of *I*^2^ was performed on the included literature: a fixed effect model was used in the absence of statistical heterogeneity (*P* > 0.1, *I*^2^ ≤ 50%); otherwise (*P* < 0.1, *I*^2^ ≥ 50%), a random effects model was used. The *Z*-test was used to test the combined effect. A difference was considered of statistical significance at *P* < 0.05 and of great statistical significance at *P* < 0.01. At the same time, the funnel chart was constructed to assess the presence of bias.

## 3. Results

The included studies involved a total of 1467 cases, of which 872 had LCA preservation and 595 did not.

### 3.1. Data Extraction and Quality Evaluation

**Figure 1:**
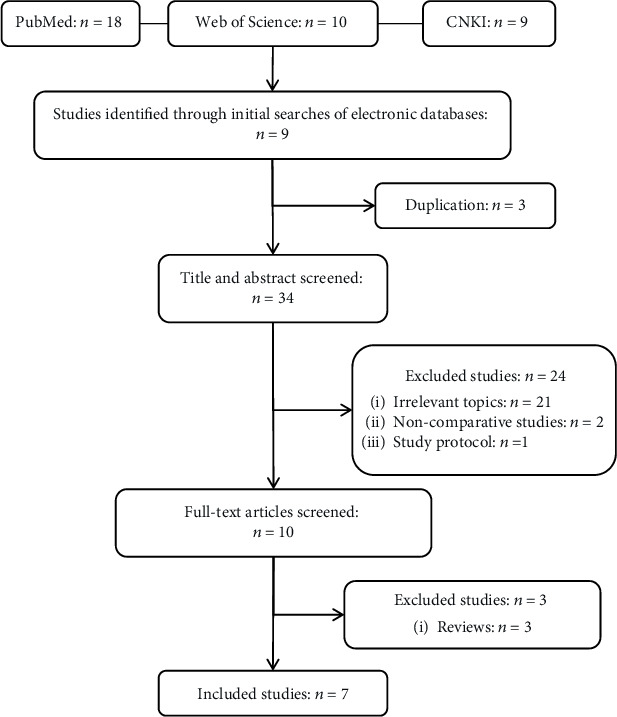
Flow diagram of studies identified, included, and excluded.

**Figure 2:**
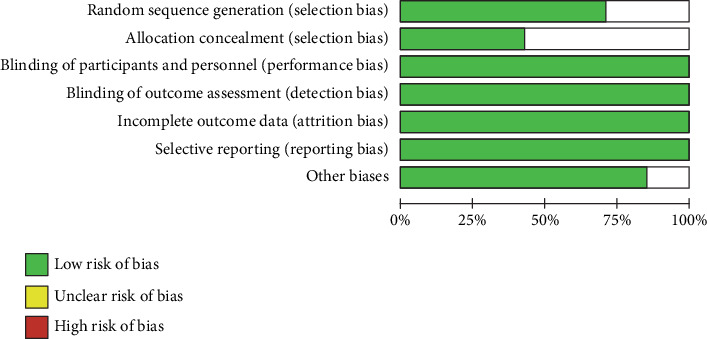
Characteristics of included studies.

The literature retrieval and screening process is shown in Figure 1. All quality evaluations were conducted using the Cochrane risk-of-bias tool for the assessment of the randomization method, allocation concealment, blinding, completeness of outcome data, selective reporting, and other biases. The final literature quality evaluation is shown in Table 1 and Figure 2.

### 3.2. Main Meta-analysis Results (Primary Outcomes)

#### 3.2.1. Duration of Surgery

**Figure 3:**
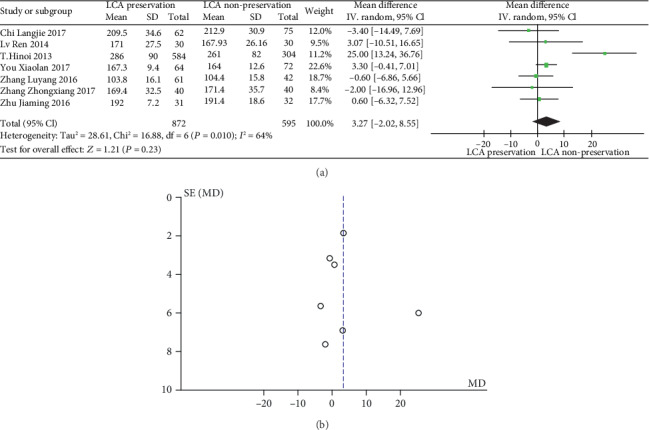
(a) Forest plot of duration of surgery; (b) funnel plot of duration of surgery.

The meta-analysis results are shown in Figures 3(a) and 3(b). The results of duration of surgery in patients with or without preservation of LCA were significantly heterogeneous (heterogeneity test: *I*^2^ = 64%, *P* = 0.010); when combined with WMD using the random effects model, the combined effect of WMD is 3.27 (95%CI = [−2.02, 8.55], *Z* = 1.21, and *P* = 0.23), which is not significantly different. Based on the results of the analysis, it can be concluded that there is no significant difference in the WMD in the duration of surgery between LCA-preserved patients and LCA-nonpreserved ones. Therefore, whether the LCA is preserved during the laparoscopic rectal cancer resection does not affect the duration of surgery. The funnel plot shows a symmetrical shape, indicating that there is no bias.

#### 3.2.2. Intraoperative Blood Loss

**Figure 4:**
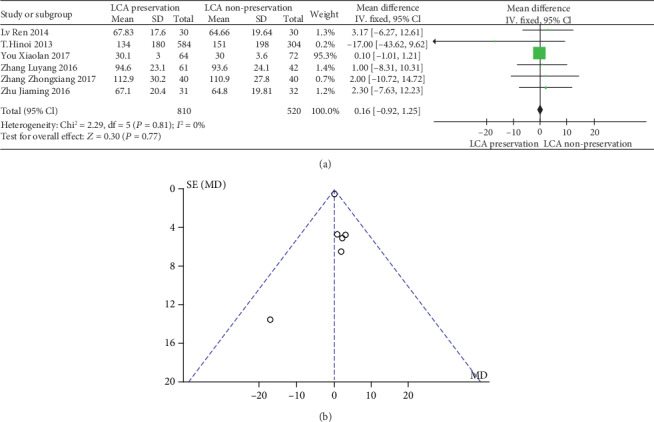
(a) Forest plot of intraoperative blood loss; (b) funnel plot of intraoperative blood loss.

The meta-analysis results are shown in Figures 4(a) and 4(b). The results of blood loss in patients with or without preservation of LCA were not significantly heterogeneous (heterogeneity test: *I*^2^ = 0%, *P* = 0.81); when combined with WMD using the fixed effects model, the combined effect of WMD is 0.16 (95%CI = [−6.27, 12.61], *Z* = 0.30, and *P* = 0.77), which is not significantly different. Based on the results of the analysis, it can be concluded that there is no significant difference in the WMD in the intraoperative blood loss between LCA-preserved patients and LCA-nonpreserved ones. Therefore, whether the LCA is preserved during the laparoscopic rectal cancer resection does not affect the amount of blood loss. The funnel plot shows a symmetrical shape, indicating that there is no bias.

#### 3.2.3. Number of Dissected Lymph Nodes

**Figure 5:**
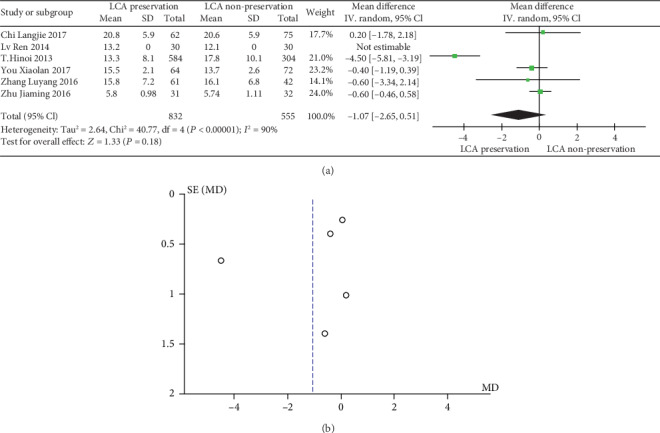
(a) Forest plot of number of dissected lymph nodes; (b) funnel plot of number of dissected lymph nodes.

The meta-analysis results are shown in Figures 5(a) and 5(b). The results of the number of dissected lymph nodes in patients with or without preservation of LCA were significantly heterogeneous (heterogeneity test: *I*^2^ = 90%, *P* ≤ 0.00001); when combined with WMD using the random effects model, the combined effect of WMD is -1.07 (95%CI = [−2.65, 0.51], *Z* = 1.33, and *P* = 0.18), which is not significantly different. Based on the results of the analysis, it can be concluded that there is no significant difference in the WMD in the number of dissected lymph nodes between LCA-preserved patients and LCA-nonpreserved ones. Therefore, whether the LCA is preserved during the laparoscopic rectal cancer resection does not affect the number of dissected lymph nodes. The funnel plot shows a symmetrical shape, indicating that there is no bias.

#### 3.2.4. Anastomotic Leakage

**Figure 6:**
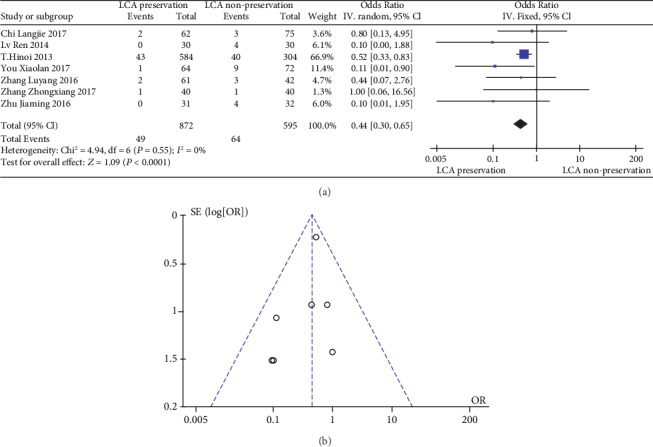
(a) Forest plot of anastomotic leakage; (b) funnel plot of anastomotic leakage.

The meta-analysis results are shown in Figures 6(a) and 6(b). The results of anastomotic leakage in patients with or without preservation of the LCA were not significantly heterogeneous (heterogeneity test: *I*^2^ = 0%, *P* = 0.55); when combined with OR using the fixed effects model, the combined effect of OR is 0.44 (95%CI = [0.30, 0.65], *Z* = 4.09, and *P* < 0.0001), which is significantly different. Based on the results of the analysis, it can be concluded that there is a significant difference in the OR of anastomotic leakage between LCA-preserved patients and LCA-nonpreserved ones. The “diamond” representing the combined effect falls to the left of the ineffective line. The patients with preservation of the LCA had a lower incidence of anastomotic leakage than those without preservation of the LCA. Therefore, preservation of the LCA can reduce the incidence of anastomotic leakage. At the same time, the funnel plot shows a symmetrical shape, indicating that there is no bias.

### 3.3. Secondary Outcomes

#### 3.3.1. Time for Intestinal Function Recovery

**Figure 7:**
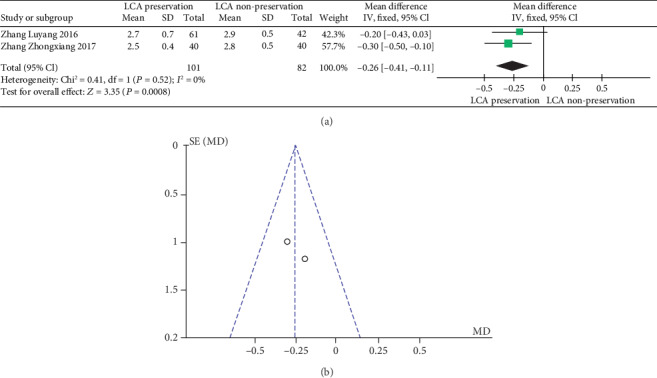
(a) Forest plot of time for intestinal function recovery; (b) funnel plot of time for intestinal function recovery.

The meta-analysis results are shown in Figures 7(a) and 7(b). The results of time for intestinal function recovery in patients with or without preservation of the LCA were not significantly heterogeneous (heterogeneity test: *I*^2^ = 0%, *P* = 0.52); when combined with WMD using the fixed effects model, the combined effect of WMD is -0.26 (95%CI = [−0.41, −0.11], *Z* = 3.35, and *P* = 0.0008), which is significantly different. Based on the results of the analysis, it can be concluded that there is a significant difference in the WMD of time for intestinal function recovery between LCA-preserved patients and LCA-nonpreserved ones. The “diamond” representing the combined effect falls to the left of the ineffective line. The patients with preservation of the LCA had shorter time for intestinal function recovery than those without preservation of the LCA. Therefore, preservation of the LCA can help patients in terms of time for intestinal function recovery. At the same time, the funnel plot shows a symmetrical shape, indicating that there is no bias.

#### 3.3.2. Postoperative Urinary and Sexual Dysfunction

**Figure 8:**
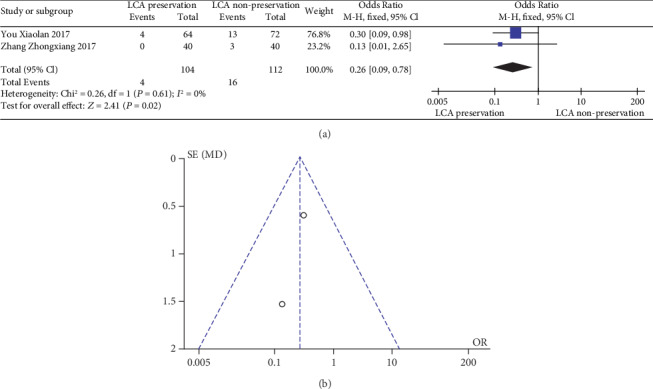
(a) Forest plot of postoperative urinary and sexual dysfunction; (b) funnel plot of postoperative urinary and sexual dysfunction.

The meta-analysis results are shown in Figures 8(a) and 8(b). The results of urinary and sexual dysfunction in patients with or without preservation of the LCA were not significantly heterogeneous (heterogeneity test: *I*^2^ = 0%, *P* = 0.61); when combined with OR using the fixed effects model, the combined effect of OR is 0.26 (95%CI = [0.09, 0.78], *Z* = 2.41, and *P* = 0.02), which is significantly different. Based on the results of the analysis, it can be concluded that there is a significant difference in the OR of postoperative urinary and sexual dysfunction between LCA-preserved patients and LCA-nonpreserved ones. The “diamond” representing the combined effect falls to the left of the invalid line. The patients with preservation of the LCA had a lower incidence of postoperative urinary and sexual dysfunction than those without preservation of the LCA. Therefore, preservation of the LCA can reduce the potential of urinary and sexual dysfunction. At the same time, the funnel plot shows a symmetrical shape, indicating that there is no bias.

#### 3.3.3. Postoperative Hospital Stay

**Figure 9:**
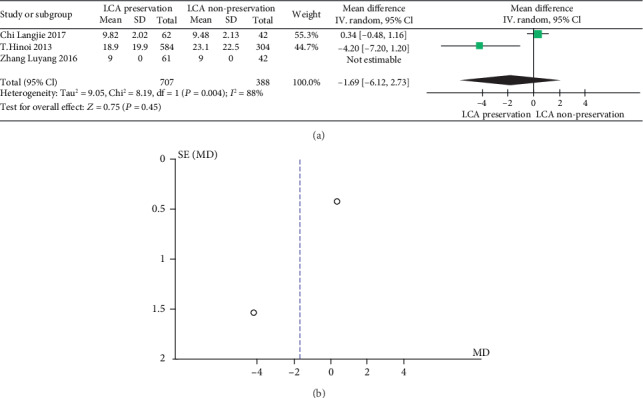
(a) forest plot of postoperative hospital stay; (b) funnel plot of postoperative hospital stay.

The meta-analysis results are shown in Figures 9(a) and 9(b). The results of postoperative hospital stay in patients with or without preservation of the LCA were significantly heterogeneous (heterogeneity test: *I*^2^ = 88%, *P* = 0.004); when combined with WMD using the random effects model, the combined effect of WMD is -1.69 (95%CI = [−6.21, 2.73], *Z* = 0.75, and *P* = 0.45), which is not significantly different. Based on the results of the analysis, it can be concluded that there is no significant difference in the WMD of postoperative hospital stay between LCA-preserved patients and LCA-nonpreserved ones. Therefore, whether the LCA is preserved during the laparoscopic rectal cancer resection does not affect the postoperative hospital stay. The funnel plot shows a symmetrical shape, indicating that there is no bias.

## 4. Discussion

There has been a long debate about whether to preserve the left colonic artery in TME under laparoscopy. So far, there is no clear consensus. Anastomotic leakage is one of the most serious complications after rectal cancer surgery. Anastomotic blood supply and tension are two important factors affecting the incidence of anastomotic leakage. Theoretically, preservation of the LCA can improve the blood supply to the colon [14, 15]; there are studies to detect the pressure of the marginal artery by using an instrument during surgery [16]. It is concluded that preservation of the LCA can improve the blood perfusion in the colon. In this meta-analysis, comprehensive clinical data shows that preservation of the LCA can reduce postoperative anastomotic leakage. Some scholars believe that high ligation of the inferior mesenteric artery may increase the probability of pelvic autonomic nerve injury, which leads to genitourinary dysfunction [17, 18], but other scholars suggest that accurate localization of the gap can minimize the probability of pelvic autonomic nerve injury. There is no significant relationship between pelvic autonomic nerve injury and ligation level of the artery [19]. In the present study, statistical data showed that the patients with preservation of the LCA had a lower incidence of urinary and sexual dysfunction and shorter time to venting than those without preservation of the LCA. However, since the sample size of this study was only 319, it may be deduced that preserving the LCA can reduce the risk of pelvic autonomic nerve injury, while further study is necessary to justify the conclusion.

## 5. Conclusions

The evidence from the current study suggests that preservation of the LCA during laparoscopic rectal cancer resection shows a significant effect on reducing the incidence of anastomotic leakage and postoperative urinary and sexual dysfunction, as well as the time for intestinal function recovery, but the effect on reducing duration of surgery, amount of blood loss, number of dissected lymph nodes, and postoperative hospital stay was not significant. Therefore, this surgical method can be recommended in the clinical practice. However, our conclusion still needs to be tested by more data in the future studies.

## Figures and Tables

**Table 1:** Characteristics of included studies.

Study	Year	Country	Adequate sequence generation	Allocation concealment	Blinding	Incomplete outcome data addressed	Free of selective reporting	Free of other biases
Hinoi et al. [7]	2013	Japan	Yes	Yes	Yes	Yes	Yes	No
Zang et al. [8]	2016	China	Unclear	Yes	Yes	Yes	Yes	No
You et al. [9]	2017	China	Unclear	Unclear	Yes	Yes	Yes	No
Zhu et al. [10]	2016	China	Yes	Unclear	Yes	Yes	Yes	No
Zhang and Zhang [11]	2017	China	Yes	Unclear	Yes	Yes	Yes	No
Lv et al. [12]	2014	China	Yes	Unclear	Yes	Yes	Yes	Unclear
Chi et al. [13]	2017	China	Yes	Yes	Yes	Yes	Yes	No

## Data Availability

The retrospective data used to support the findings of this study are included within the article.
